# Tlx3 Exerts Direct Control in Specifying Excitatory Over Inhibitory Neurons in the Dorsal Spinal Cord

**DOI:** 10.3389/fcell.2021.642697

**Published:** 2021-04-29

**Authors:** Filipe A. Monteiro, Rafael M. Miranda, Marta C. Samina, Ana F. Dias, Alexandre A. S. F. Raposo, Patrícia Oliveira, Carlos Reguenga, Diogo S. Castro, Deolinda Lima

**Affiliations:** ^1^Unidade de Biologia Experimental, Departamento de Biomedicina, Faculdade de Medicina da Universidade do Porto, Porto, Portugal; ^2^Pain Research Group, Instituto de Biologia Molecular e Celular, Porto, Portugal; ^3^Instituto de Investigação e Inovação em Saúde, Universidade do Porto, Porto, Portugal; ^4^Instituto de Ciências Biomédicas Abel Salazar, Universidade do Porto, Porto, Portugal; ^5^Molecular Neurobiology Group, Instituto Gulbenkian de Ciência, Oeiras, Portugal; ^6^Instituto de Medicina Molecular João Lobo Antunes, Faculdade de Medicina da Universidade de Lisboa, Lisboa, Portugal; ^7^Diagnostics, Institute of Molecular Pathology and Immunology, University of Porto, Porto, Portugal; ^8^Stem Cells & Neurogenesis Group, Instituto de Biologia Molecular e Celular, Porto, Portugal

**Keywords:** T-cell leukemia homeobox 3, dorsal horn, spinal cord, excitatory neuron, chromatin immunoprecipitation

## Abstract

The spinal cord dorsal horn is a major station for integration and relay of somatosensory information and comprises both excitatory and inhibitory neuronal populations. The homeobox gene Tlx3 acts as a selector gene to control the development of late-born excitatory (dILB) neurons by specifying glutamatergic transmitter fate in dorsal spinal cord. However, since Tlx3 direct transcriptional targets remain largely unknown, it remains to be uncovered how Tlx3 functions to promote excitatory cell fate. Here we combined a genomics approach based on chromatin immunoprecipitation followed by next generation sequencing (ChIP-seq) and expression profiling, with validation experiments in *Tlx3* null embryos, to characterize the transcriptional program of Tlx3 in mouse embryonic dorsal spinal cord. We found most dILB neuron specific genes previously identified to be directly activated by Tlx3. Surprisingly, we found Tlx3 also directly represses many genes associated with the alternative inhibitory dILA neuronal fate. In both cases, direct targets include transcription factors and terminal differentiation genes, showing that Tlx3 directly controls cell identity at distinct levels. Our findings provide a molecular frame for the master regulatory role of Tlx3 in developing glutamatergic dILB neurons. In addition, they suggest a novel function for Tlx3 as direct repressor of GABAergic dILA identity, pointing to how generation of the two alternative cell fates being tightly coupled.

## Introduction

The dorsal horn of the spinal cord modulates and conveys peripheral somatosensory input related to pain, itch, touch, cold, and warm to higher brain centres. This is accomplished by a heterogeneous neuronal population composed of various subtypes of relay neurons and interneurons (Lima and Coimbra, 1986; Caspary and Anderson, 2003; Helms and Johnson, 2003; Todd, 2010; Haring et al., 2018). The correct generation of excitatory and inhibitory neurons is crucial in eliciting accurate physiological responses to somatosensory events, and disturbing the balance between these two cell populations has been associated with the pathological perception of pain (Scholz and Woolf, 2002; Yekkirala et al., 2017).

Knowledge on the cellular and molecular developmental mechanisms governing cell fate choice and cell subtype specification is central for understanding neuronal diversity and is being currently used for deciphering somatosensory circuits at the dorsal spinal cord (Prescott et al., 2014; Hernandez-Miranda et al., 2017). Most of the neurons that settle in the superficial dorsal horn are generated during the late phase of dorsal neurogenesis [embryonic day (E) 12 to E13.5] from a common pool of progenitors expressing the transcription factors Gsx1/2 and Ascl1 (Gross et al., 2002; Muller et al., 2002; Mizuguchi et al., 2006; Wildner et al., 2006). From these progenitors, two related subpopulations arise in a salt and pepper manner: GABAergic dILA neurons characterized by the expression of Ptf1a, Lbx1, Pax2, Lhx1/5, and Gbx1 homeodomain (HD) transcription factors (Gross et al., 2002; Muller et al., 2002; Glasgow et al., 2005; John et al., 2005; Pillai et al., 2007), and glutamatergic dILB neurons, which co-express Tlx1/3, Lbx1, Lmx1b, Prrxl1, and Brn3a (Cheng et al., 2004, 2005; Xu et al., 2008; Rebelo et al., 2010). In the *Tlx1/3* knockout mice, prospective dILB neurons are transformed into Pax2^+^-dILA cells (Cheng et al., 2004), whereas in *Ptf1a* knockout mice, prospective dILA neurons switch into Tlx3^+^-dILB cells (Glasgow et al., 2005), with the resulting cells expressing, in both cases, subtype specific, terminal differentiation markers. Altogether, these observations indicate that Ptf1a and Tlx3 (partially redundant with Tlx1) function as selector genes of both GABAergic dILA and glutamatergic dILB neurons, respectively. Importantly, they also suggest that the mechanisms providing the two neuronal subtypes are intertwined, with specification of either dlLA or dlLB identity occurring at the expenses of the alternative cell fate. In support of this is the fact that, in dILA neurons, Ptf1a promotes Pax2, Lhx1, and Lhx5 expression while repressing Tlx3 and Lmx1b (Glasgow et al., 2005). Conversely, in dILB neurons, Tlx3 promotes the expression of Prrxl1, Lmx1b, and Brn3a/Pou4f1 (Qian et al., 2002; Xu et al., 2008) and antagonizes Lbx1-mediated specification of GABAergic phenotype (Cheng et al., 2005).

Although advances have been made in the identification of transcriptional regulators involved in specifying both classes of dorsal late-born interneurons (Cheng et al., 2004, 2005; Glasgow et al., 2005; Mizuguchi et al., 2006; Wildner et al., 2006), little is known on the underlying molecular mechanisms, in particular the associated transcriptional networks. In the present study, we investigate Tlx3 direct gene regulatory actions in the context of its regulatory function in dorsal spinal cord neurogenesis (Cheng et al., 2004, 2005). Using chromatin immunoprecipitation followed by next generation sequencing (ChIP-seq) of Tlx3 and gene expression profiling of the developing dorsal spinal cord, we generated a comprehensive list of Tlx3 direct target genes, which were further validated in *Tlx3* null embryos. We found Tlx3 to activate genes with a wide spectrum of functions involved in the early specification and later differentiation aspects of glutamatergic dILB neurons. Surprisingly, we also found Tlx3 to work as a repressor of many GABAergic genes. We conclude that the role of Tlx3 as a selector gene of excitatory dILB neurons involves direct and opposite regulation of genes involved in dILB and dILA specification and differentiation.

## Materials and Methods

### Animals

The animals used in this study were maintained in accordance with the European Union Directive 2010/63/EU, national Decreto-lei n°113-2013, and the protocols described were approved by the IBMC Ethical Committee and by the Portuguese Veterinarian Board. NMRI and C57BL/6 mouse strains were bred and housed at the i3S animal facility, under temperature-, and light-controlled conditions. The embryonic day 0.5 (E0.5) was the midday of the vaginal plug.

### Chromatin Immunoprecipitation

Tlx3 chromatin immunoprecipitation (ChIP) assays using either dorsal or ventral spinal cord tissue from NMRI mouse embryos at E14.5 were performed as previously described (Regadas et al., 2014). For gene target validation, one Tlx3 peak for each locus within a shortest distance from the TSS was selected. ChIP-qPCR validation was performed using the following primers: Lmx1b TGTGTGAGGAATATCAATGGAGT and GG GAGACAGCCAGTGCTTA; Prrxl1 TTATGCGCCATTAGACT TGC and CTCTCTGCCTGGGTGAAAAT; Pou4f1 GCCTCA GATTTCCACTCCAT and GAAGGCTCCACTTCATCACC; Tl x3 TTTCCGCTGCTAATTCCTCT and ATTTCGGGTTTGA GAAGCTG; Slc17a6 GCTGCCTTATGCCACCAT and CGGTC CCTTGGTACATCATT; Npy1r TATCTCCAGACCCCAGAG GA and GCCTACAGCAGAAGTGGACA; Cck ACCGCTGCTA TTGCCTTAGT and ACCCTGTCCTTCCTTCCTCT; Grin3a T CCTGCATGTGGTAGTTTGG and GCAAGGCAATGAGAATA GCA; Zic1 CTTTTGCGGTTTATCTTCCTG and GGTGTC GTCCTTTCAATTCAT; Trpc3 TGATGGCTTAATTTCCCC TAA and GCCCTGCTTCATTCTCACTT; Robo2 CAGC AATTTAGTGAGAGCCAAT and GGAATCAAGTCCAGATG TTTCA; Lphn2 AGCGAGGCTAACGAGAAGG and CTT GCCTGCATTGATGATTT; Gpr26 GAGAGGGGAGTGGGG TTAAT and AAGCTCTAAGCGGATGCTTT; Zic2 CGGC CCTATGAATATGAACA and TTTGTTGCAGCTTTTCTTGG; Pax2 GCCGTTTATCTCTCCTTCCA and GGTTCCCCAGCT ACAGTCTC; Lhx1 CCGCAGTACCATTGTCTTCA and TTTT TGCTACATCCCCCAAT; Lhx5 ACGAGTTGTCAGCGAAACC and ATTCATCTCCCTCCCGTTC; Gbx1GAGCCATTCACA CAATCACC and CCAGCGTTCTCATCTCGTT; Slc32a1 TTCA CTAAGGGGGAGTTGGT and ACCAGCACAACATGCAAAC; Grik3 GTTCCTTGAGGCCATGTTTC and TCGACTGG GGACCTTTTAGA; Sall3 TCAAATCGCCAATCACCTTA and TCCCCAGCTCATCACAAATA; Dll1 (ORF1) GTCTCAGGA CCTTCACAGTAG and GAGCAACCTTCTCCGTAGTAG; and, Ppp1r9a (ORF2) GCAGCCGAAAATGAGAAAGT and TCGATCCAGTAGCTCTCCAA.

### ChIP-Seq

The product of several ChIP assays was pooled in order to generate enough material for library preparation, which was quantified with Picogreen (Invitrogen). Libraries were prepared from 10 ng of immunoprecipitated DNA according to standard Illumina ChIP-seq protocol and sequenced with Illumina HiSeq2000. Raw reads were mapped to the mouse genome (NCBI37/mm9) with Bowtie version 0.12.7 (Langmead et al., 2009), and PCR duplicates removed with SAMTools (Li et al., 2009). Peaks were called with MACS 1.4.1 (Zhang et al., 2008) with *P*-value cut-off at 10^–5^. Subsampling confirmed that peak calling saturation was achieved with approximately 90% of sequenced reads. Peaks were annotated to the nearest transcription start site (TSS) using Peak Analyzer 1.4 (Salmon-Divon et al., 2010). Tlx3 peak at genomic coordinates chr8: 69213987-69215025 was re-annotated from *Npy5r* to *Npy1r*, as *Npy5r* is not expressed at E14.5 mouse dorsal spinal cord and *Npy1r* expression has been shown to be Tlx3-dependent (Guo et al., 2012). Sequencing data has been deposited at ArrayExpress^1^ with the accession number E-MTAB-6974. To retrieve Tlx3 binding profile in selected genomic segments, Tlx3 ChIP-seq data set was loaded into UCSC Genome Browser^2^ (Waterston et al., 2002) and the ENCODE ChIP-seq data sets for H3K4me1, H3K4me3, and H3K27ac from E14.5 neural tube and the conservation across 30 vertebrates species analyzed.

### Gene Expression Profiling

Dorsal spinal cords dissected from litters of E14.5 wild type embryos of unknown sex were processed for RNA extraction using Trizol and RNeasy Mini kit (Qiagen) extraction procedures. Five replicates of wild type embryos were analyzed, each sample with tissue pooled from three embryos. Expression profiling was done using Affymetrix Mouse Gene 1.0 ST Arrays at the Gene Expression Unit of Instituto Gulbenkian de Ciência. Data analyses were performed using R and Bioconductor. Statistical analysis was performed using False Discovery Ratio (FDR) with *P*-value < 0.05. Following common criteria in the field, a log2 expression average of biological replicates ≥7 was used to determine expressed genes. Expression profiling data has been deposited at ArrayExpress (see text footnote 1) with the accession number E-MTAB-9963.

### Motif Searches and Gene Ontology

*De novo* search for enriched DNA motifs within 20 bp of Tlx3 peak summits was performed using CisFinder software^3^ (Sharov and Ko, 2009) with default parameters, using equivalent 3 kb upstream genomic regions as background control. To search for co-enriched motifs in the vicinity of Tlx3 binding events, CisFinder search was performed using 200 bp genomic regions centered at peak summits, upon masking of the Tlx3 motif. Searches for transcription factor motif similarities was performed against mouse motif database using Tomtom algorithm^4^ (Gupta et al., 2007) with default parameters. Genomic distribution of Tlx3 peaks was performed using ChIPpeakAnno. Gene annotation was performed according to the nearest TSS allowing for a maximum distance of 2.14 Mb and considering ENSEMBL coding genes only. Functional annotation and enrichment for gene ontology (GO) terms was determined using DAVID 6.8 version^5^ (Huang da et al., 2009). Terms with a *P*-value < 5.0 × 10^–2^ and a Functional Annotation Clustering Enrichment Score >2.0 were considered enriched. For Biological Processes, clustering was performed, and representative terms from each cluster are shown. For clarity, clusters of generic terms were not displayed.

### Immunofluorescence and *in situ* Hybridization

Immunofluorescence was performed as previously described (Rebelo et al., 2010). The guinea pig anti-Tlx3 and rabbit anti-Lmx1b antibodies (1:1000 dilution) were a gift from Tomas Müller (Max-Delbrück-Centrum for Molecular Medicine, Germany). For *in situ* hybridization, *Tlx3* null and control embryos kindly provided by Dr. Leping Cheng (Shanghai Institutes for Biological Sciences, China) were fixed in 4% PFA/PBS at 4°C for 48 h, and then placed in 30% sucrose/PBS overnight. Embryos were embedded in OCT compound (Surgipath) and frozen before sectioning in a cryostat (Leica). Embryos serial sections (10 μm thick) were collected in a way that cervical, thoracic and lumbar axial levels were represented in each microscope slide. Each axial level was carefully mapped for each embryo using an Atlas of Mouse Development. Cryosections were processed for *in situ* hybridization as previously described (Casarosa et al., 1999). Probes were synthesized by *in vitro* transcription of PCR fragments containing a T7 site and amplified in two consecutive rounds using cDNA from E14.5 mouse dorsal spinal cord tissue. Primers used included a T7 priming sequence (GGTAATACGACTCACTATAGGG) in reverse and the following sequences: Lphn2/Adgrl2 GCTTCTGTACCAACCCCAGA and CACTGGCAGCGTCT CTATCA, Gpr26 CTTCTGACCCCTTCGTGTATTC and TT TGGGTTACAGCAGCAAACA, Zic2 GGGCACCTTAGGATCG TCTTAT and GAAAAAGAAAAGGCCCATCAC, Lhx1 GGA TGAAACAGCTAAGCGCG and GCTGACATGGAGTGGAG AGG, Lhx5 CCAAAGAACGCCGCATGAAA and TTCG TTGAGCTCAGGGTTGG, Sall3 CCTCAGTACAGCTTCAGG and CTGATGTTGGTACAGTGGG. *Robo2* (Sabatier et al., 2004) and *Gbx1* (John et al., 2005) probes were generated from plasmids provided by Mark Tessier-Lavigne (Genentech, California) and Stefan Britsch (Ulm University, Germany), respectively. For double staining, *in situ* hybridization was followed by immunofluorescence with a few modifications. Briefly, C57BL/6 mouse embryo tissue sections were incubated with guinea pig anti-Tlx3 antibody (1:50 dilution) for two days at 4°C. Fluorescent and bright-field z-stack images were captured on a Zeiss Axio Imager Z1 microscope with a Plan-Apochromat 63×/1.40 Oil DIC objective, with a step size of 0.27 μm. The acquired fluorescent z-stack images were deconvolved using Huygens professional 19.10 software. *In situ* signals, using one selected *z*-plane image, were converted into a red pseudocolor and merged with the fluorescent signals of the matching *z*-plane image, using Fiji/ImageJ software (Schindelin et al., 2012).

### Cell Culture

ND7/23 cell line was from the European Collection of Cell Cultures. This cell line was cultured and transfected as previously described (Monteiro et al., 2014) with a Tlx3 expressing vector (Regadas et al., 2013).

### RNA Extraction and Real-Time Quantitative PCR

RNA extraction and real-time qPCR assays were performed essentially as previously described (Monteiro et al., 2014), with the following modifications: (i) RNA was purified using total RNA isolation kit (NZYTech), including a step of DNase I treatment of the binding column, and cDNA was prepared using oligo dT primers and M-MuLV Reverse Transcriptase (NZYTech) according to the manufacturer’s instructions; (ii) real-time qPCR analysis was performed using the NZYSpeedy qPCR Green Master Mix (NZYTech) on a CFX384 qPCR System (Bio-Rad). The primer sequences were CCTGACAAAGAAGCGCCTTA and ACACGTTTGGGCAAAAGTACA for *Tlx3* targeting the 3′UTR, and TTGCTGACCTGCTGGATTAC and GTCCTTTTCACCAGCAAGC for *Hprt* housekeeping gene. Expression of endogenous *Tlx3* transcript was normalized to the control gene *Hprt*.

### Statistical Analysis

For gene expression profiling of E14.5 dorsal spinal cord, see respective section above. For *in situ* hybridizations, the number of E14.5 mouse embryos used ranged from *n* = 3 to *n* = 7 in wild type and *n* = 3 to *n* = 4 in *Tlx3* knockout mice, being the exact number for each experiment depicted in the figure legends. ChIP-qPCR experiments, on E14.5 chromatin, were performed at least twice with independent samples and one representative experiment is shown. The results are plotted as mean ± standard deviation (SD), and significance determined by Student’s *t*-test. The statistical significance of the overlap of gene lists was determined using a hypergeometric distribution calculator available at http://nemates.org/MA/progs/overlap_stats.html, using the estimated number of genes in the Affymetrix microarray. The representation factor is the number of overlapping genes divided by the expected number of overlapping genes drawn from two independent groups, with a representation factor >1 indicating a greater overlap than expected by chance. RT-qPCR results are shown as the mean of triplicates ± SD of two independent experiments. Student’s *t*-test statistical analysis was used to determine statistical significance between cells transfected with empty vector (vector) and cells transfected with mouse Tlx3.

## Results

### Characterization of Tlx3 Transcriptional Program in Embryonic Dorsal Spinal Cord

**FIGURE 1:**
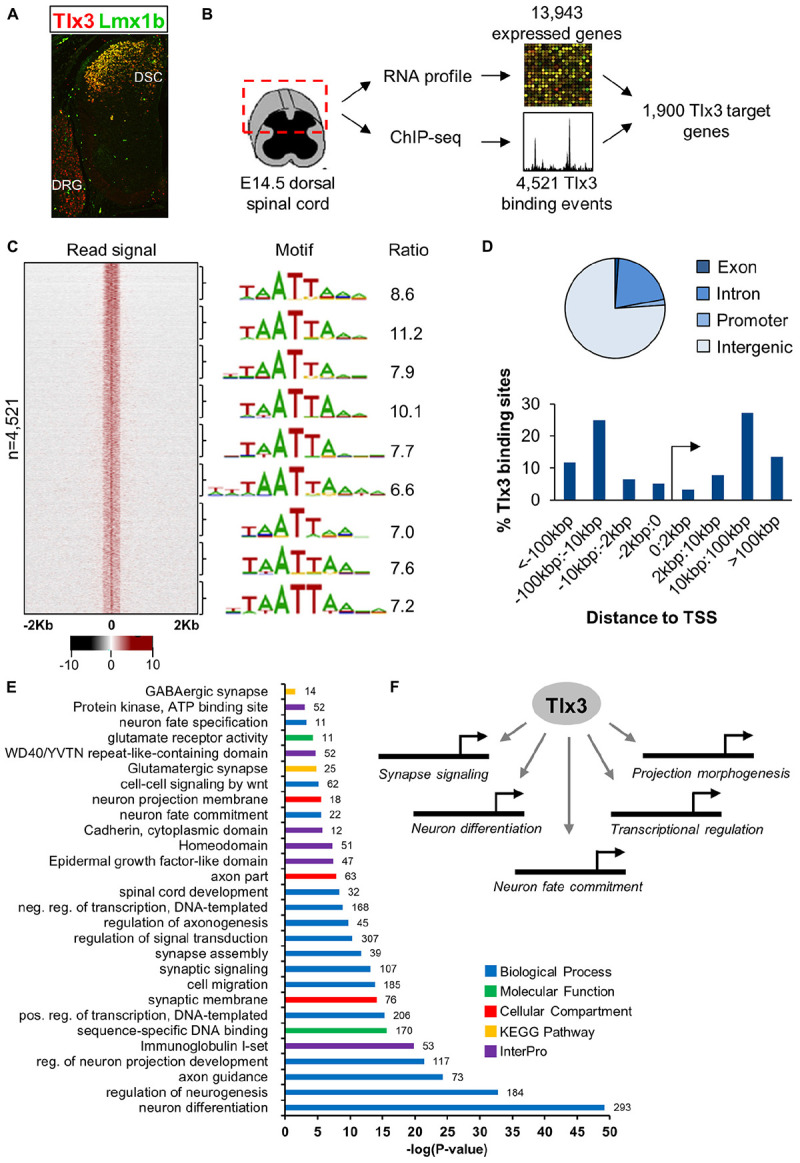
Characterization of Tlx3 transcriptional program in embryonic dorsal spinal cord. **(A)** Immunofluorescence analysis of Tlx3 and Lmx1b in E14.5 mouse spinal cord. Expression of Tlx3 and Lmx1b extensively co-localize in dorsal spinal cord, whereas in dorsal root ganglion only Tlx3 is detected. **(B)** Strategy used to identify direct targets of Tlx3 in dorsal spinal cord. **(C)** Heat map displays sequencing signal intensity in genomic regions centered at Tlx3 peak summits, ordered top-to-bottom by increasing *P*-value (left). Highest enriched motif in each bin of 500 bound regions (middle). Enrichment ratio of each motif matches in bound sequences over upstream control sequences (right). **(D)** Genomic features associated with Tlx3 binding sites (top) and relative association to the nearest TSS (bottom). **(E)** Biological processes over-represented in the list of Tlx3 putative target genes (i.e., bound and expressed in the dorsal spinal cord) using DAVID GO analysis. Colored bars represent the GO category, KEGG pathway or InterPro protein class and in front of each bar is the number of target genes associated with that term. **(F)** Summary of the functions controlled by Tlx3 in the dILB neurons specification.

To understand how Tlx3 acts as a selector gene in the specification of the glutamatergic populations of dorsal horn neurons, we began by defining the repertoire of genes directly regulated by Tlx3 by combining genome-wide mapping of Tlx3 binding sites with expression profiling of dorsal spinal cord region. For that, we performed ChIP-seq using chromatin extracted from mouse dorsal spinal cord at embryonic day 14.5 (E14.5), a stage at which Tlx3 is highly expressed in newly born postmitotic neurons (Figure 1A). This resulted in a list of 4,521 binding sites (*P* < 10^−5^) (Supplementary Table 1 and Figure 1B), which shows consistent enrichment in a bin-by-bin analysis for the expected Tlx3 consensus binding motif TAATTA (Figure 1C). Binding motif is composed by a HD half-site (i.e., TAAT or ATTA) overlapping a second half-site, resembling the consensus binding sequence for Tlx3 family members previously identified by *in vitro* approaches (Berger et al., 2008; Noyes et al., 2008; Pujato et al., 2014). Most of the Tlx3 target sites (∼70%) fall within intergenic regions located at distances greater than 10 kb from the nearest TSS, suggesting preferential binding to distal regulatory elements (Figure 1D). Association of binding events to putative target genes was performed so as to maximize the statistical significance of the overlap between Tlx3 “bound” genes and a list of genes expressed in E14.5 dorsal spinal cord (Supplementary Table 2 and see section “Materials and Methods” for details). Accordingly, 4,521 Tlx3 binding sites (*P* < 10^−5^) are associated with 2,773 putative target genes, 1,900 of which are expressed in dorsal spinal cord region (Supplementary Table 3 and Figure 1B). In line with a large proportion of the detected Tlx3 binding events being regulatory, the comparison with the genome-wide profiles of histone marks obtained from whole neural tube at the same developmental stage provided by ENCODE (Gorkin et al., 2020) revealed a strong signal of histone 3 lysine 4 monomethylation (H3K4me1) and H3K4 trimethylation (H3K4me3) (Supplementary Figure 1), which in combination with H3K27 acetylation (H3K27ac) mark active enhancers or promoters, respectively (Shlyueva et al., 2014). In addition, we also found the group of Tlx3 bound genes to significantly overlap with that of genes expressed in dorsal spinal cord (representation factor: 1.4; *P* < 3.493e-113), again supporting the biological relevance of our Tlx3 genome-wide binding data set.

Next, we performed GO analyses to identify terms overrepresented in the list of Tlx3 target genes (i.e., genes bound and expressed in the dorsal spinal cord) using DAVID (Huang da et al., 2009). Consistent with Tlx3 known function in the specification and subsequent differentiation of glutamatergic dorsal horn precursor neurons, we found enrichment of terms derived from different GO categories, KEGG pathways and InterPro protein families related to neuron fate commitment, differentiation, projection development and migration (Supplementary Tables 4–6 and Figures 1E,F). Of particular notice were genes encoding transcription factors (namely of the HD family) with a known function in “spinal cord development,” related to glutamatergic differentiation (e.g., *Prrxl1* and *Isl1*) and, surprisingly, also to GABAergic differentiation (e.g., *Lhx1* and *Gbx1*) (discussed below). In addition, several genes were related to synapse signaling and assembly, suggesting a role for Tlx3 also in later steps of neuronal differentiation. Altogether, these data indicate that Tlx3 regulates distinct aspects of spinal dorsal horn neurogenesis, including early steps of neuronal sub-type specification and later processes of neuronal maturation (Figure 1F).

### Tlx3 Directly Promotes Glutamatergic Differentiation

**FIGURE 2:**
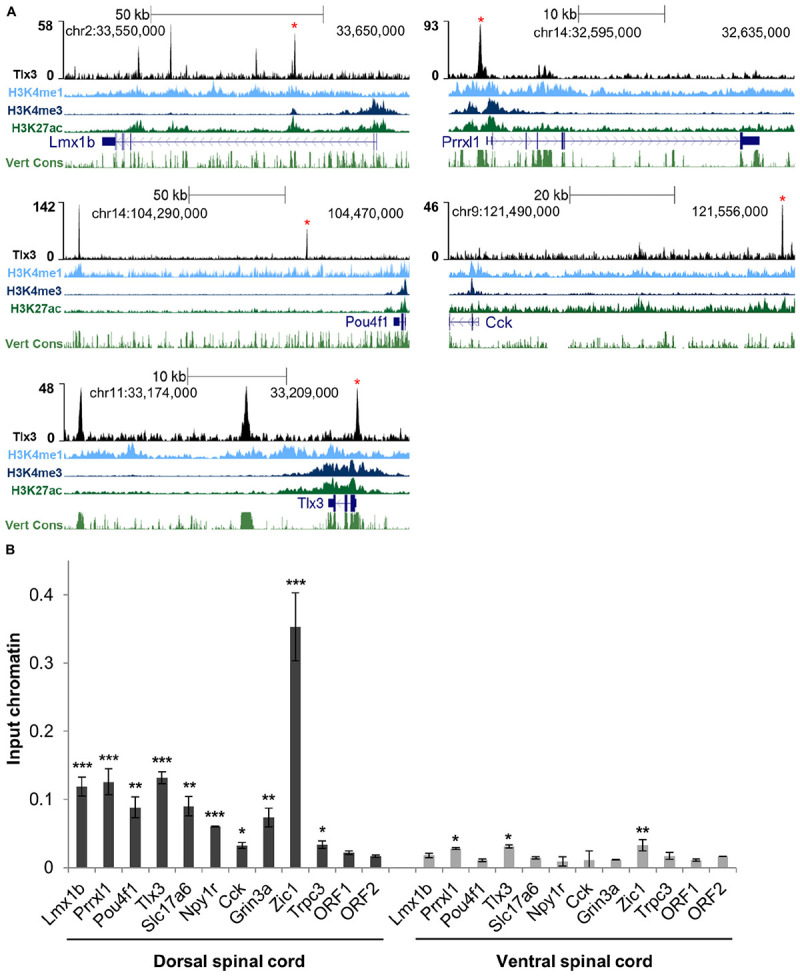
Tlx3 directly controls the expression of transcription factors and terminal differentiation genes involved in glutamatergic differentiation. **(A)** Tlx3 binding profile (in black) within genomic regions spanning target genes. Target gene structure and direction of transcription (blue), H3K4me1 (light blue), H3K4me3 (blue), and H3K27ac (green), as well as multispecies vertebrate conservation (green) plots are shown. ENCODE annotations are from histone marks of ChIP-seq data sets using E14.5 mouse neural tube (Gorkin et al., 2020). Data tracks extracted using the UCSC genome browser (Waterston et al., 2002). Binding sites validated by ChIP-qPCR are marked by red asterisks. **(B)** Validation of Tlx3 binding sites by independent ChIP-qPCR using chromatin extracted from either E14.5 dorsal or ventral spinal cords. Two negative control regions (ORF1/2) are shown. Mean ± SD; *P < 0.05, **P < 0.01, and ***P < 0.001 as compared to ORF2 with Student’s *t*-test; *n* = 3 experimental replicates.

To assess whether Tlx3 binding is associated with the regulation of excitatory differentiation markers, we charted the Tlx3 targets encoding for transcription factors and terminal differentiation molecules that were reported in the literature as Tlx3-dependent genes. Analysis of null mice for *Tlx* genes identified a set of transcription factor genes specifically expressed in dILB but not in dILA spinal dorsal horn neurons in a Tlx1/3 or Tlx3 dependent manner. These include *Lmx1b*, *Prrxl1/Drg11*, and *Pou4f1/Brn3a* (Qian et al., 2002; Xu et al., 2008; Zou et al., 2012), being *Lmx1b* and *Prrxl1* involved in dorsal spinal cord morphogenesis (Chen et al., 2001; Ding et al., 2004; Rebelo et al., 2010) and *Pou4f1* required for the early wave of neuropeptide *Tac1* expression (Xu et al., 2008). Strikingly, all these genes were associated with Tlx3 binding events identified by ChIP-seq and subsequently validated by ChIP-qPCR from dorsal spinal cord chromatin (but not from ventral spinal cord chromatin as expected, given that Tlx3 is not expressed in that region) (Figures 2A,B). Noteworthy, the enrichment of histone marks at Tlx3 peaks at those gene loci were indicative of Tlx3 occupancy at active *cis*-regulatory elements (Figure 2A). In addition, Tlx3 was recruited to its own locus at multiple sites (Figures 2A,B), suggesting an auto-regulatory feedback loop. Overexpression of Tlx3 in ND7/23 neuronal cell line strongly repressed the expression of endogenous *Tlx3* mRNA (Supplementary Figure 2), suggesting autorepression as a control mechanism of *Tlx3* transcription, a feature previously described for *Prrxl1* (Monteiro et al., 2014).

Genetic ablation in mouse embryos has also previously shown that Tlx1/3 positively regulate the expression of the vesicular glutamate transporter *Slc17a6/Vglut2* (Fremeau et al., 2001), as well as a set of synaptic transmission genes characteristic of the glutamatergic phenotype, such as genes encoding for neuropeptides *Grp*, *Tac1*, *Cck*, *Sst*, *Adcyap1* and *Nts*; neuropeptide receptors *Grpr*, *Galr1*, *Npy1r*, and *Tacr1*; glutamate receptors *Gria2*, *Gria3*, *Grin3a*, and *Grin3b*; GABA receptors *Gabra1*, *Gabra5*, and *Gabrb2*, and signaling molecule *Prkcg/PKC gama* (Cheng et al., 2004; Li et al., 2006; Xu et al., 2008, 2013; Guo et al., 2012). Strikingly, we found prominent Tlx3 binding to a large number of these genes, namely *Slc17a6*, *Cck*, *Adcyap1*, *Nts*, *Galr1*, *Npy1r*, *Grin3a*, and *Gabrb2* (Supplementary Table 1 and Figures 2A,B). We did not find evidence that the Tlx3-dependent genes *Grp*, *Tac1*, *Sst*, *Grpr*, *Tacr1*, *Gria2*, *Gria3*, *Grin3b*, *Gabra1*, *Gabra5*, and *Prkcg* are under direct control of Tlx3, although we cannot exclude that this may result from a limitation of our gene annotation strategy, which considers only the nearest TSS to a Tlx3 binding event. Taken together, our results show that the Tlx3 role as a master regulator of dorsal horn glutamatergic neuronal differentiation and peptidergic transmitter phenotype acquisition is largely based on direct binding mechanisms and identifies many important examples of target genes involved.

Previous analysis of double *Tlx1/3* or *Tlx3* null mouse spinal cords by *in situ* hybridization has also revealed deregulation of genes not yet associated with glutamatergic or GABAergic neurotransmitter phenotypes. Several neuronal differentiation genes, namely the transcription factors *Ebf2, Maf/c-Maf*, and *Mafa* (Qian et al., 2002; Hu et al., 2012) and effector genes *Pcp4*, *Trpc3*, and *Enc1* (Li et al., 2006) were shown to be downregulated, and the transcription factors *Zic1* and *Zic4* (Ding et al., 2004) upregulated. Interestingly, we found Tlx3 binding to genes encoding all above mentioned transcription factors as well as *Pcp4*, *Trpc3*, and *Enc1* (Supplementary Table 1 and Figures 2A,B). These observations further highlight the complexity of the transcriptional program directly governed by Tlx3.

**FIGURE 3:**
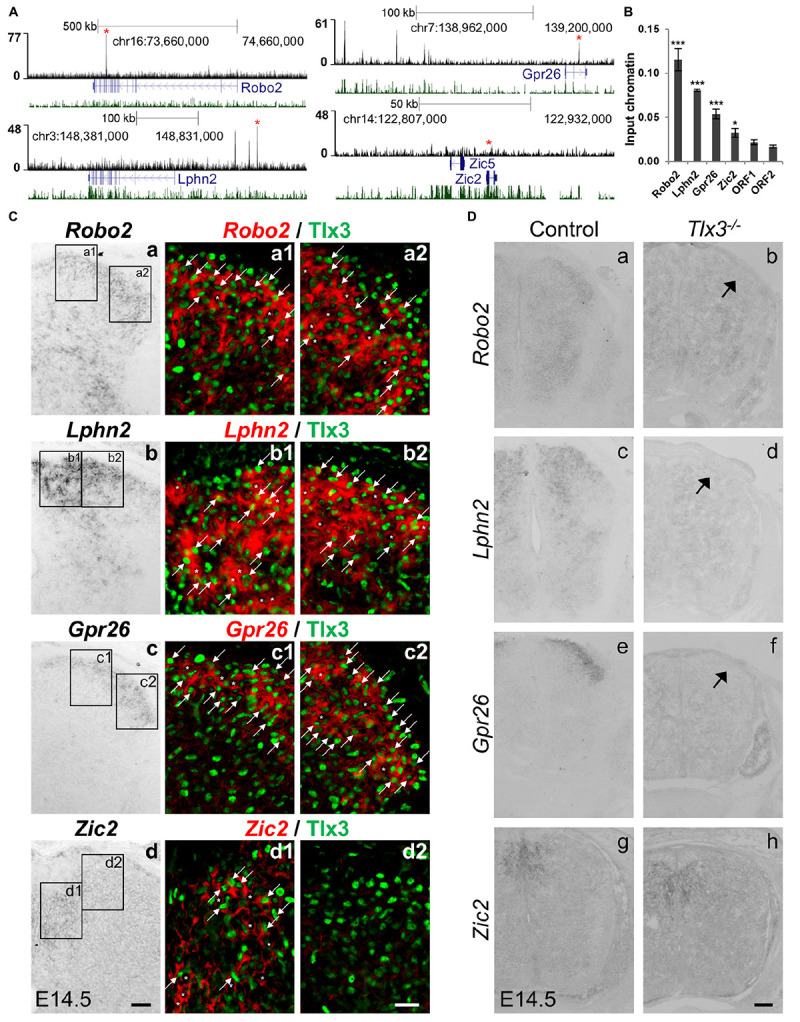
Identification of novel Tlx3 target genes. **(A)** Tlx3 binding profile (black) within genomic regions spanning target genes. Target gene structure and direction of transcription (blue) as well as multispecies vertebrate conservation plot (green) are shown. Binding sites used for ChIP-qPCR validation are marked by red asterisks. **(B)** Validation of Tlx3 binding sites by independent ChIP-qPCR using chromatin extracted from E14.5 dorsal spinal cords. Two negative control regions (ORF1/2) are shown. Mean ± SD; *P < 0.05 and ***P < 0.001 as compared to ORF1 with Student’s *t*-test; *n* = 3 experimental replicates. **(C)** Expression of *Robo2*, *Lphn2*, *Gpr26*, and *Zic2* in Tlx3^+^ neurons. a–d *In situ* hybridizations using transverse sections of lumbar spinal cord of E14.5 wild type embryos. a1–d2 Double staining of Tlx3 protein (a1–2, b1–2, c1–2, d1–2, green) with either *Robo2* (a1–2, red), *Lphn2* (b1–2, red), *Gpr26* (c1–2, red), and *Zic2* (d1–2, red) mRNA. Note that bright-field *in situ* hybridization signals were converted into red pseudocolor signals. Note the extensive co-expression of *Robo2*, *Lphn2*, and *Gpr26* with Tlx3 (a1–2, b1–2, c1–2, arrows), but little co-expression of *Zic2* with Tlx3. Expression of putative target genes in Tlx3^–^ cells is labeled an asterisk. Scale bars: a–d 50 μm; a1–d2 20 μm. **(D)** Reduction or loss of the expression of target genes in *Tlx3* null dorsal spinal cord assessed by *in situ* hybridization. a–h Transverse sections through the lumbar spinal cord of E14.5 wild type [a (*n* = 5); c (*n* = 4); e (*n* = 6); g (*n* = 4)] and *Tlx3* null mutants [b (*n* = 3); d (*n* = 3); f (*n* = 3); h (*n* = 3)]. Expression of *Robo2*, *Lphn2*, and *Gpr26* was markedly reduced (b, d, f, arrow). *Zic2* expression was not affected (h), as previously observed (Ding et al., 2004). Scale bar: 100 μm.

Beyond ascertaining whether known Tlx3-depentent genes were under Tlx3 direct regulation, we also probed our gene list to identify novel direct targets whose expression has not yet been shown to depend on Tlx3. With that aim, we focused on four genes: three associated with at least one highly significant (top 10% lowest *P*-value) Tlx3 binding event and with enriched expression in superficial dorsal horn (*Robo2*, *Lphn2*, and *Gpr26*), as determined by the analysis of expression patterns in the literature and public *in situ* hybridization database GenePaint^6^ (Li et al., 2006; Eichele and Diez-Roux, 2011); and one not associated with Tlx3 binding (*Zic2*) to function as negative control (Figure 3A). These Tlx3 binding events were validated through ChIP-qPCR (Figure 3B). To verify whether these novel target genes are expressed in Tlx3^+^-cells, we performed double-staining experiments for Tlx3 protein and transcript of putative target genes, combining immunofluorescence with mRNA *in situ* hybridization, respectively. We found that, in contrast to *Zic2*, *Robo2*, *Lphn2*, and *Gpr26* are extensively expressed in Tlx3^+^-cells across the superficial dorsal horn (Figure 3C, arrows). In addition, these targets were also expressed in few Tlx3^–^-cells (Figure 3C, asterisks). During spinal cord development, Tlx1 and Tlx3 redundancy is restricted to cervical and thoracic levels, whereas at the lumbar level solely Tlx3 is expressed (Qian et al., 2002; Cheng et al., 2004, 2005). Therefore, we used lumbar spinal cord cross sections from E14.5 embryos to test whether the expression of any of these genes was altered in *Tlx3* null mice. In the spinal cord dorsal horn of *Tlx3* knockout embryos, a marked reduced expression of *Roundabout homolog 2 (Drosophila)* (*Robo2*), *Latrophilin 2* (*Lphn2*), and *G protein-coupled receptor 26* (*Gpr26*) was found, as compared to wild type controls (Figure 3D, arrows), which is in line with the observed broad expression of these target genes in Tlx3^+^-cells. By contrast, the expression of *Zic2* was not changed between both conditions. While *Robo2* is a well-known axonal pathfinding and neuronal migration receptor molecule (Jaworski et al., 2010), the roles of G protein-coupled receptors *Lphn2* and *Gpr26* in the dorsal spinal cord have not yet been determined. *Lphn2* has been implicated in postsynaptic target recognition in the hippocampus (Anderson et al., 2017) and *Gpr26* is apparently related to anxiety and depression associated behaviors (Zhang et al., 2011).

### Tlx3 Directly Suppresses GABAergic Differentiation

**FIGURE 4:**
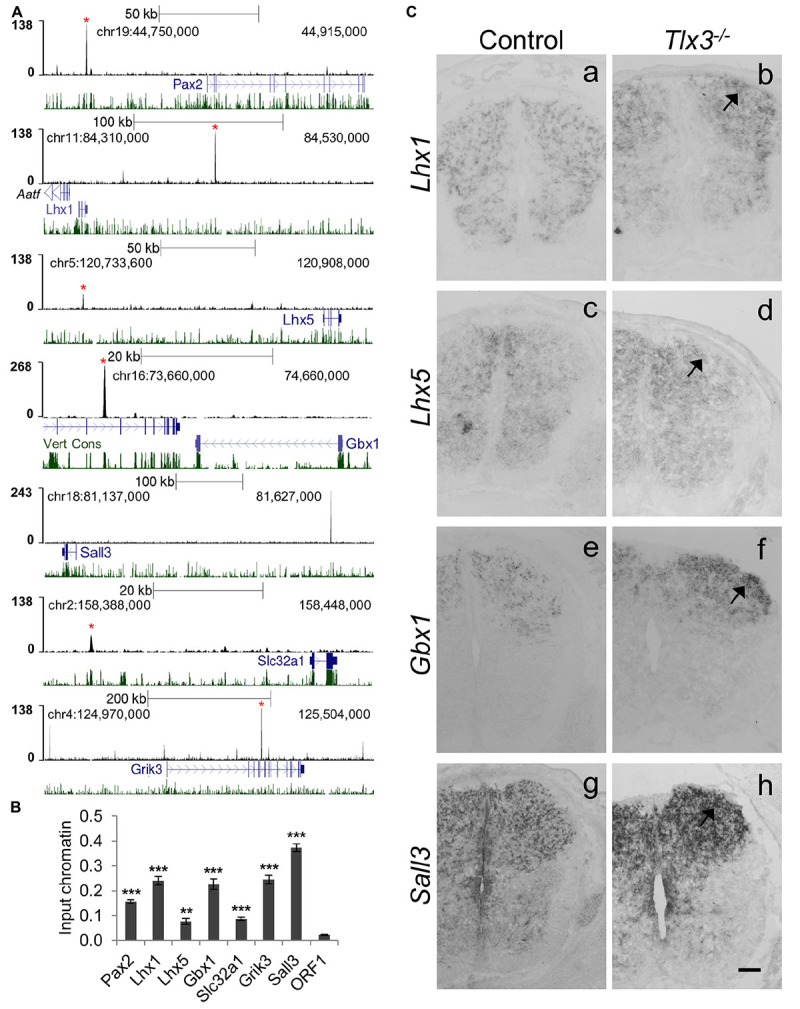
Tlx3 directly controls the expression of transcription factors and terminal differentiation genes involved in GABAergic differentiation. **(A)** Tlx3 binding profile (black) within genomic regions spanning target genes. Target gene structure and direction of transcription (blue) as well as multispecies vertebrate conservation plots (green) are shown. Binding sites validated by ChIP-qPCR are marked by red asterisks. **(B)** Validation of Tlx3 binding sites by independent ChIP-qPCR using chromatin extracted from E14.5 dorsal spinal cords. Negative control region (ORF1) is shown. Mean ± SD; **P < 0.01 and ***P < 0.001 as compared to ORF1 with Student’s *t*-test; *n* = 3 experimental replicates. **(C)** Derepression of GABAergic markers in *Tlx3* null dorsal spinal cord assessed by *in situ* hybridization. a–h Transverse sections through the lumbar spinal cord of E14.5 wild type [a (*n* = 6); c (*n* = 6); e (*n* = 7); g (*n* = 3)] and *Tlx3* null mutants [b (*n* = 4); d (*n* = 3); f (*n* = 3); h (*n* = 3)]. *Lhx1*, *Lhx5*, *Gbx1*, and *Sall3* expression in the dorsal horn was markedly increased (b, d, f, h, arrow) in *Tlx3* mutants as compared to controls. Scale bar: 100 μm.

A close inspection of genes associated with Tlx3 binding events revealed many potential targets associated with dILA neuronal development, suggesting a putative direct role of Tlx3 as suppressor of GABAergic cell fate. To investigate this, we validated Tlx3 binding to GABAergic differentiation genes by ChIP-qPCR and compared their expression in wild type versus *Tlx3* null embryos. Tlx3 binding sites were found associated with *Pax2*, *Lhx1*, *Lhx5*, and *Gbx1* homeobox genes and *Sall3*, a zinc finger transcription factor encoding gene (Figures 4A,B). In *Tlx3* mutants, the expression of *Lhx1*, *Lhx5*, *Gbx1*, and *Sall3* transcripts was markedly increased in the dorsal horn (Figure 4C, arrows). *Sall3* was recently shown to be exclusively expressed in GABAergic neurons of the dorsal horn (Haring et al., 2018) and is important for terminal differentiation of olfactory glomerular interneurons (Harrison et al., 2008). These data suggest that Tlx3 suppresses GABAergic genetic program in excitatory glutamatergic neurons, by directly binding to homeobox genes that promote the inhibitory neuron identity.

**FIGURE 5:**
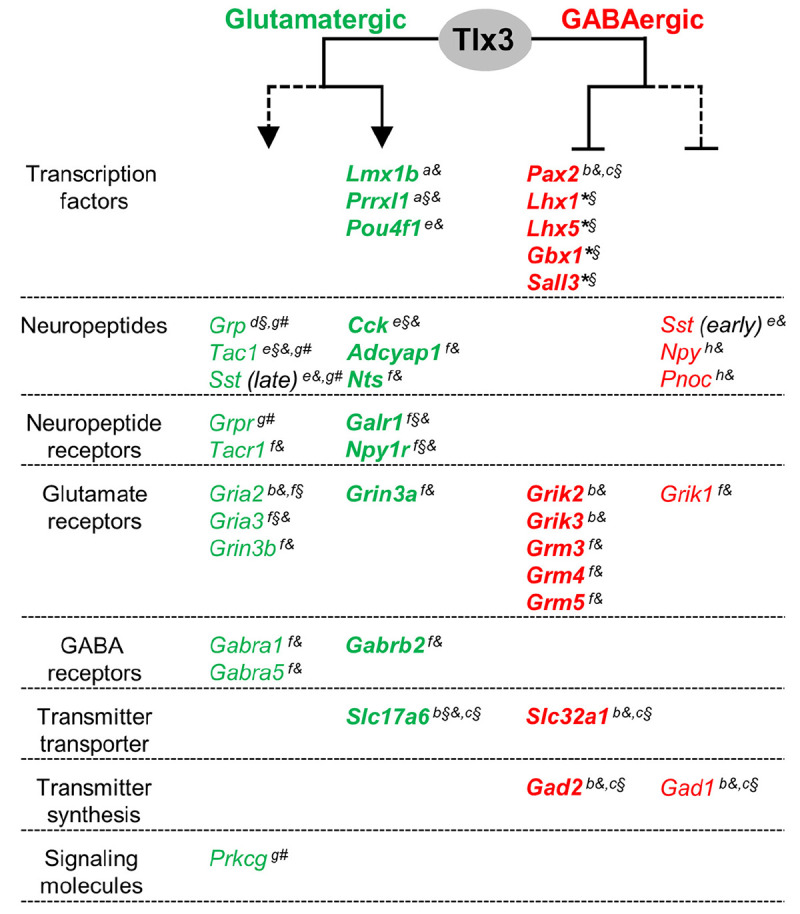
A model for a dual activity of Tlx3 in promoting glutamatergic, while concomitantly suppressing GABAergic specification program in dILB neurons. Tlx3 direct target genes include transcription factors, neuropeptides, neuropeptide and glutamate and GABA receptors, and molecules for neurotransmitter transport. Genes expressed in glutamatergic cells, most of which are Tlx3^+^/Pax2^–^-cells, are markedly reduced or eliminated in *Tlx3* knockout mice, whereas genes expressed in GABAergic cells, most of which are Tlx3^–^/Pax2^+^-cells, are upregulated in *Tlx3* knockout mice. Expression data was retrieved from the following reported studies: Qian et al., 2002 (a), Cheng et al., 2004 (b), Cheng et al., 2005 (c), Li et al., 2006 (d), Xu et al., 2008 (e), Guo et al., 2012 (f), Xu et al., 2013 (g), Huang et al., 2008 (h), and our study (*) using *Tlx3* single knockout (§), *Tlx1/3* double knockout (§), and/or *Tlx3* conditional knockout (#) mice. Line, direct regulation; dashed line, indirect regulation; arrow, activation of expression; blunt line, inhibition of expression.

Previous studies have also found the expression of generic markers of GABAergic differentiation to be upregulated in the dorsal spinal cord of *Tlx1/3* null embryos. This is the case of *Gad1/2* and *Slc32a1/Viaat*, involved, respectively, in GABA synthesis and transport (Cheng et al., 2004), and genes encoding for neuropeptides *Sst* (early expression wave), *Npy* and *Pnoc*, and glutamate receptors *Grik1*, *Grik2*, *Grik3*, *Grm3*, *Grm4*, and *Grm5* genes (Cheng et al., 2004; Huang et al., 2008; Xu et al., 2008; Guo et al., 2012), all terminal differentiation genes mainly expressed in spinal dorsal horn GABAergic neurons. Strikingly, of all the above genes, we found Tlx3 binding to *Slc32a1*, *Grik2*, *Grik3*, *Grm3*, *Grm4*, *Grm5*, and *Gad2* (Supplementary Table 1 and Figures 4A,B). Altogether these results suggest that Tlx3 suppresses GABAergic differentiation in dILB neurons at distinct levels, including transcription factors but also terminal differentiation genes with exclusive expression in dILA neurons (Figure 5).

## Discussion

Accumulating evidence shows that Tlx3 plays a pivotal role in the morphogenesis of the dorsal horn of the spinal cord, where it functions as a master regulator of the development of excitatory glutamatergic neurons (Xu et al., 2008). This role is exerted redundantly with Tlx1 where both factors are co-expressed, namely at more rostral regions (Cheng et al., 2004). Several Tlx3-dependent genes were previously identified in mouse dorsal spinal cord through gene targeting studies in mice (Qian et al., 2002; Cheng et al., 2004, 2005; Li et al., 2006; Xu et al., 2008, 2013; Guo et al., 2012). However, understanding how this protein functions has been compromised by the lack of data on direct molecular interactions. In this study, we bridged this gap by providing the first genome-wide characterization of Tlx3 transcriptional program in the developing nervous system. This expanded the list of Tlx3 targets, representing an important resource for future studies.

Our study argues that Tlx3 activates a broad and complex transcriptional program in the differentiation of late-born glutamatergic dILB neurons, while repressing genes associated with the alternative GABAergic dILA phenotype. This dual activity is in line with evidence showing that mechanisms specifying dILA and dILB cell lineages, which are derived from the same progenitor pool (Gross et al., 2002; Muller et al., 2002), are tightly coupled. Accordingly, while loss of determinants of one lineage (e.g., Tlx1/3, Gsx1/2, Ascl1, Ptf1a, and Lbx1) results in conversion into the alternative phenotype (Cheng et al., 2004, 2005; Glasgow et al., 2005; Mizuguchi et al., 2006), their forced expression is sufficient, in some cases, to cause a neurotransmitter cell fate switch (Mizuguchi et al., 2006; Wildner et al., 2006; Hori et al., 2008). These loss- and gain-of-function studies support the idea that each of these lineage precursors has the plasticity to differentiate into the alternative phenotype.

Virtually all genes known to be specifically expressed in Tlx3^+^ glutamatergic neurons, including transcription factors and terminal effector genes, are dependent on Tlx3, since their expression is markedly reduced or eliminated in *Tlx3* knockout mice (Qian et al., 2002; Cheng et al., 2004, 2005; Li et al., 2006; Xu et al., 2008, 2013; Guo et al., 2012). Thus, Tlx3 appears to be at the head of the differentiation of superficial dorsal horn glutamatergic neurons. Strikingly, our results suggest that most of these transcription factors, as well as many of the terminal effector genes, are in fact under the direct control of Tlx3 (Figure 5). In line with such master control function, an important group of the here identified direct targets of Tlx3 encodes for transcriptional regulators such as Lmx1b, Prrxl1, and Pou4f1/Brn3a (Qian et al., 2002; Xu et al., 2008), whose combinatorial and dynamic expression is likely to generate various subpopulations of mature excitatory dorsal horn neurons (Rebelo et al., 2010). In addition, our work suggests that Tlx3 also activates downstream effectors of glutamatergic phenotype (Brohl et al., 2008; Guo et al., 2012; Xu et al., 2013), similarly to the model proposed for Ptf1a in dILA lineage specification (Guo et al., 2012; Borromeo et al., 2014).

The present results indicate that Tlx3 transactivates genes associated with dILB identity, which is in line with the previous observation that Tlx3 physically interacts with the transcriptional coactivator cyclic adenosine monophosphate (cAMP)-response element-binding protein (CREB)-binding protein (CBP), and that this interaction is necessary for the expression of glutamatergic neuronal subtype markers in differentiating embryonic stem cells (Shimomura et al., 2015). Although a role for Tlx3 in transcriptional repression has been less documented, several lines of evidence suggest this activity may rely on the interaction with cofactors of the Gro/TLE family: (i) Tlx closely related paralogs (Tlx1, Tlx2, and Tlx3) contain a conserved Engrailed homology 1 (Eh1)-like motif (present in most HD proteins) that mediates the recruitment of Gro/TLE corepressors, which have been implicated in the patterning of ventral neuronal subtypes (Muhr et al., 2001); (ii) Tlx1 and Tlx3 were shown to physically interact with TLE1 (Riz et al., 2009); and (iii) Tlx1–TLE1 interaction is necessary for Tlx1 transcriptional regulation of target genes (Riz et al., 2009, 2010).

Tlx3 gene ablation results also in supernumerary dILA neurons, suggesting the concomitant regulation of both alternative cell fates (Cheng et al., 2004, 2005). Previously, it has been speculated that Tlx3 may repress the dILA neuronal fate (while promoting dILB identity) via mechanism based on protein-protein interactions with other important determinant Lbx1 (Cheng et al., 2005). Our observations open an alternative pathway whereby Tlx3 binds to and directly represses not only GABAergic differentiation factors (i.e., *Pax2*, *Lhx1/5*, and *Gbx1*), but also terminal effector GABAergic genes (i.e. *Grik2/3*, *Grm3/4/5*, and *Slc32a1*) (Figure 4, and Cheng et al., 2004; Cheng et al., 2005; Guo et al., 2012). In the current study, the biological significance of Tlx3 binding to dILA and dILB genes is based on gene expression analysis of *Tlx3* knockout mice. As in these mice glutamatergic dILB precursors are transformed into GABAergic dILA neurons, we cannot exclude the possibility that the gene expression changes observed are an indirect consequence from this fate conversion. Nevertheless, the fact that Tlx3 ectopic expression in developing chick dorsal spinal cord results in robust glutamatergic differentiation at the expenses of expression of GABAergic marker genes in presumptive GABAergic cells (Cheng et al., 2004) argues in favor of Tlx3 playing direct regulatory roles.

An important question that remains to be addressed is how can Tlx3 function simultaneously as an activator and repressor in the same cellular context. Examples of other HD factors with similar dual activities include Pbx1 and Chx10, during midbrain and spinal cord development, respectively (Clovis et al., 2016; Villaescusa et al., 2016). In the latter case, it was shown that Chx10 HD protein promotes the expression of spinal V2a interneuron genes, while concomitantly suppressing the expression of motor neuron genes by acting as a DNA-binding competitor of the Isl1-Lhx3-HD complex (Clovis et al., 2016). It is tempting to speculate that similar mechanisms may be at play in the case of Tlx3, although this remains to be further investigated. In any case, both activation and repression are associated with direct DNA binding and most likely result from differences at regulatory regions targeted by Tlx3. One possibility is that the binding of Tlx3 to variants of its consensus binding motif could result in Tlx3 adopting distinct protein structures leading to the recruitment of co-factors with distinct activities. Alternatively, activation versus repression could rely on the combinatorial action of Tlx3 with distinct groups of transcription factors that co-occupy the same gene regulatory regions. Although not conclusively, none of these hypotheses was supported by our *de novo* search for enriched DNA motifs at the vicinity of Tlx3 binding events (data not shown).

In conclusion, our data strongly indicate that Tlx3 may concomitantly promote and repress gene expression, biasing dILB precursors to become excitatory glutamatergic neurons while suppressing the alternative inhibitory GABAergic cell fate (Figrue 5). This dual transcriptional activity of a HD selector gene may be of particular importance in developmental contexts, when segregation of related differentiated cell types takes place from a common pool of progenitors.

## Data Availability Statement

Datasets presented in this study can be found in ArrayExpress with accession numbers E-MTAB-6974 (Tlx3 ChIP-seq from mouse E14.5 dorsal spinal cord) and E-MTAB-9963 (Transcription profiling of mouse E14.5 dorsal spinal cord).

## Ethics Statement

The animal study was reviewed and approved by the IBMC Ethical Committee and by the Portuguese Veterinarian Board.

## Author Contributions

FM, CR, DC, and DL conceived and designed the research. FM, RM, MS, and AD performed the research. FM, AR, PO, CR, DC, and DL analyzed the data. FM, DC, and DL wrote the manuscript and supervised the work. All authors read and approved the final manuscript.

## Conflict of Interest

The authors declare that the research was conducted in the absence of any commercial or financial relationships that could be construed as a potential conflict of interest.

## Footnotes

1 https://www.ebi.ac.uk/arrayexpress/

2 http://genome-euro.ucsc.edu/

3 http://lgsun.grc.nia.nih.gov/CisFinder/

4 https://meme-suite.org/meme/tools/tomtom

5 http://david.abcc.ncifcrf.gov/

6 https://gp3.mpg.de/

## Supplementary Material

Supplementary Figure 1 Pattern of H3K4me1, H3K4me3, and H3K27ac histone marks around Tlx3 peak summits in E14.5 mouse neural tube (DOCX 1461 kb).

Supplementary Figure 2 Overexpression of Tlx3 induces repression of the endogenous Tlx3 expression in ND7/23 cells (DOCX 675 kb).

Supplementary Table 1 Genomic coordinates of Tlx3 binding sites determined by ChIP-Seq (XLSX 448 kb).

Supplementary Table 2 Genes expressed in E14.5 mouse dorsal spinal cord (XLSX 391 kb).

Supplementary Table 3 List of Tlx3 target regions associated to genes that are expressed in embryonic dorsal spinal cord (XLSX 369 kb).

Supplementary Table 4 Overrepresented gene ontology biological process categories associated with Tlx3 target gene (XLSX 326 kb).

Supplementary Table 5 Overrepresented gene ontology KEGG pathway terms associated with Tlx3 target gene (XLSX 25 kb).

Supplementary Table 6 Overrepresented gene ontology InterPro terms associated with Tlx3 target genes (XLSX 46 kb).
