# Supplementary material for: Tlx3 Exerts Direct Control in Specifying Excitatory Over Inhibitory Neurons in the Dorsal Spinal Cord
Source: Front Cell Dev Biol. 2021 Apr 29;9:642697. doi: 10.3389/fcell.2021.642697 (PMC8117147; doi:10.3389/fcell.2021.642697)
Supplement: Supplementary Figure 1 — Pattern of H3K4me1, H3K4me3, and H3K27ac histone marks around Tlx3 peak summits in E14.5 mouse neural tube (DOCX 1461 kb). [file Table_7.DOCX]

Supplementary Material


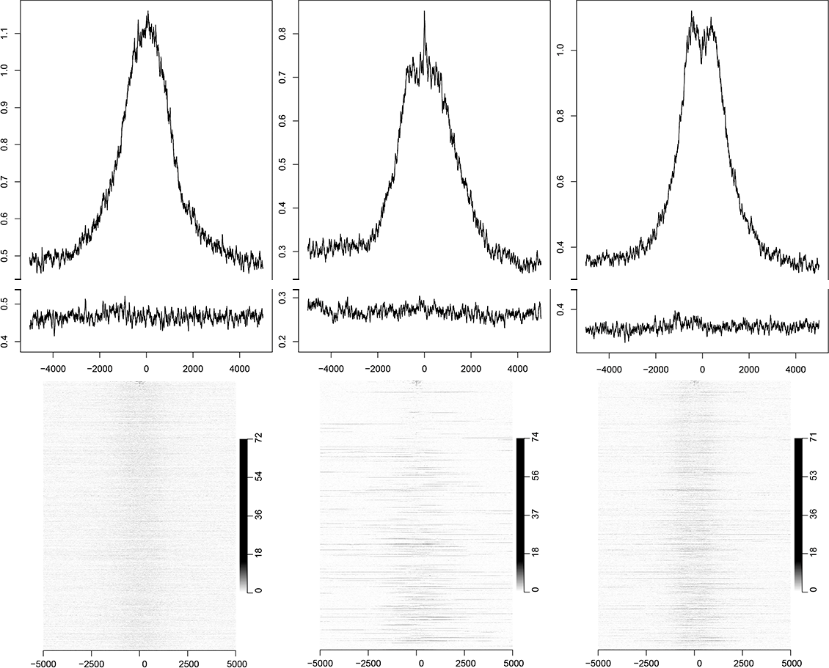


**H3K4me1**

**Distance to peak summits (bp)**

**Average score**

**H3K4me3**

**H3K27ac**

**Supplementary Figure 1 | Pattern of H3K4me1, H3K4me3 and H3K27ac histone marks around Tlx3 peak summits in E14.5 mouse neural tube.** Upper charts show ChIP-seq enrichment profiles of H3K4me1, H3K4me3 and H3K27ac histone marks in ±5 kb genomic regions centered at Tlx3 peak summits. Bottom charts show the enrichment profile of histone marks at 10 kb upstream genomic regions, as a control. Heat maps show signal enrichment of histone modifications at Tlx3 bound regions (±5 kb centered at Tlx3 peak summits). Color scales indicate the relative signal intensity on heat maps. H3K4me1, H3K4me3 and H3K27ac histone marks are from public ENCODE ChIP-seq data in E14.5 mouse neural tube.
