# Supplementary material for: Tlx3 Exerts Direct Control in Specifying Excitatory Over Inhibitory Neurons in the Dorsal Spinal Cord
Source: Front Cell Dev Biol. 2021 Apr 29;9:642697. doi: 10.3389/fcell.2021.642697 (PMC8117147; doi:10.3389/fcell.2021.642697)
Supplement: Supplementary Figure 2 — Overexpression of Tlx3 induces repression of the endogenous Tlx3 expression in ND7/23 cells (DOCX 675 kb). [file Table_8.DOCX]

Supplementary Material


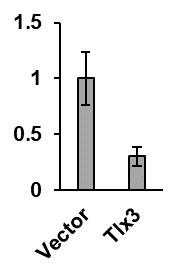


******

**Tlx3 mRNA expression**

**normalized to *Hprt***

**Supplementary Figure 2 | Overexpression of Tlx3 induces repression of the endogenous Tlx3 expression in ND7/23 cells**. ND7/23 cells were transfected with an expression construct for Tlx3 and empty vector (vector) and after 16 h the endogenous Tlx3 mRNA expression of was assessed by RT-qPCR. mRNA level intensities were normalized to Hprt housekeeping gene. Mean ± S.D.; **P < 0.01 with Student’s t-test; n = 3.
